# Identification of a prognostic gene signature based on an immunogenomic landscape analysis of bladder cancer

**DOI:** 10.1111/jcmm.15960

**Published:** 2020-10-13

**Authors:** Yongwen Luo, Liang Chen, Qiang Zhou, Yaoyi Xiong, Gang Wang, Xuefeng Liu, Yu Xiao, Lingao Ju, Xinghuan Wang

**Affiliations:** ^1^ Department of Urology Zhongnan Hospital of Wuhan University Wuhan China; ^2^ Department of Biological Repositories Zhongnan Hospital of Wuhan University Wuhan China; ^3^ Human Genetics Resource Preservation Center of Hubei Province Wuhan China; ^4^ Laboratory of Precision Medicine Zhongnan Hospital of Wuhan University Wuhan China; ^5^ Cancer Precision Diagnosis and Treatment and Translational Medicine Hubei Engineering Research Center Wuhan China; ^6^ Department of Pathology Lombardi Comprehensive Cancer Center Georgetown University Medical School Washington DC USA

**Keywords:** bladder cancer, immunogenomic landscape, overall survival, prognosis, the cancer genome atlas

## Abstract

Cancer immune plays a critical role in cancer progression. Tumour immunology and immunotherapy are one of the exciting areas in bladder cancer research. In this study, we aimed to develop an immune‐related gene signature to improve the prognostic prediction of bladder cancer. Firstly, we identified 392 differentially expressed immune‐related genes (IRGs) based on TCGA and ImmPort databases. Functional enrichment analysis revealed that these genes were enriched in inflammatory and immune‐related pathways, including in ‘regulation of signaling receptor activity’, ‘cytokine‐cytokine receptor interaction’ and ‘GPCR ligand binding’. Then, we separated all samples in TCGA data set into the training cohort and the testing cohort in a ratio of 3:1 randomly. Data set GSE13507 was set as the validation cohort. We constructed a prognostic six‐IRG signature with LASSO Cox regression in the training cohort, including AHNAK, OAS1, APOBEC3H, SCG2, CTSE and KIR2DS4. Six IRGs reflected the microenvironment of bladder cancer, especially immune cell infiltration. The prognostic value of six‐IRG signature was further validated in the testing cohort and the validation cohort. The results of multivariable Cox regression and subgroup analysis revealed that six‐IRG signature was a clinically independent prognostic factor for bladder cancer patients. Further, we constructed a nomogram based on six‐IRG signature and other clinicopathological risk factors, and it performed well in predict patients' survival. Finally, we found six‐IRG signature showed significant difference in different molecular subtypes of bladder cancer. In conclusions, our research provided a novel immune‐related gene signature to estimate prognosis for patients' survival with bladder cancer.

## 1 INTRODUCTION

Bladder cancer is one of the most common malignant cancers worldwide. The new global cancer statistics showed an estimated 549 000 new bladder cancer cases and 200 000 deaths in 2018.^1^ The main treatment of bladder cancer is surgery and chemotherapy. The therapeutic effect is still not satisfactory, Patients who underwent radical cystectomy had a 5‐year overall survival rate of 66% and a 10‐year survival rate of 43%, and there is still a lack of public recognized, universally applicable bladder cancer diagnosis and prognostic evaluation markers.

The tumour microenvironment is the comprehensive cell environment on which tumour cells depend. It consists of cellular components and non‐cellular components. The cell components incorporate tumour cells, inflammatory cells, immune cells, mesenchymal stem cells, endothelial cells and fibroblasts associated with tumours. Non‐cellular components mainly consist of cytokines, chemokines and so on, which together constitute a complex tumour microenvironment.

Increasing evidence has revealed tumour microenvironment plays an important role in carcinogenesis and progression of bladder cancer.^2^, ^3^, ^4^ Some immunological checkpoint molecules have been found to be particularly important in the regulation of tumour microenvironment, A series of corresponding immunological checkpoint inhibitors as PD‐1/PD‐L1 has been applied in bladder cancer treatment.^5^, ^6^ However, current PD‐1/PD‐L1 immunotherapies showed a poor clinical efficacy for clinical patients only range from 16% to 25%. An increasing number of studies have found that many other immune‐related genes can also affect the prognosis and efficacy of immunotherapy. Therefore, there is an urgent need to elucidate the immunophenotypes and identify novel immune‐related prognostic genes in bladder cancer.

In this study, we aimed to gain insight into the immunogenomic landscape of bladder cancer and develop an immune‐related gene signature to improve the prognostic predictions of bladder cancer.

## 2 MATERIALS AND METHODS

### 2.1 Bladder cancer gene expression data sets and immune‐related genes

Raw counts of RNA‐sequencing data for bladder cancer samples were obtained from The Cancer Genome Atlas (TCGA) data portal (https://tcga‐data.nci.nih.gov/tcga/), which consisted of 414 bladder cancer specimens and 19 adjacent non‐tumour bladder specimens. Data set GSE13507 (Illumina human‐6 v2.0 expression beadchip) of GeneChip Transcriptome Array was downloaded from Gene Expression Omnibus (GEO) database (http://www.ncbi.nlm.nih.gov/geo/). This data set included 165 primary bladder cancer samples, 23 recurrent non‐muscle invasive tumour tissues, 58 normal looking bladder mucosae surrounding cancer and 10 normal bladder mucosae for microarray analysis. We also obtained a list of immune‐related genes (IRGs) from the Immunology Database and Analysis Portal (ImmPort).^7^, ^8^

### 2.2 Data processing and differentially expressed gene (DEG) screening

For TCGA‐BLCA data, the gene expression data were based on the RNA‐sequencing technology of Illumina HiSeq. Ensembl IDs were converted to gene symbol by the corresponding GENCODE files. ‘edgeR’ R package was performed for differential expression analysis. The cut‐off criteria for screening DEGs were the FDR (false discovery rate) < 0.05 and |log_2_ (fold change)| ≥ 1. For the microarray analyses, RMA method was used to perform background correction for the raw expression data at first, and log2 transformation and normalization were performed for processed signals. Then, probes were annotated by the corresponding annotation files.

### 2.3 Pathway and functional enrichment analysis

To further indicate the underlying mechanism of IRGs impact on correlative clinical feature, the pathway enrichment analysis was performed using above differentially expressed IRGs. Metascape (http://metascape.org/) was used to gain insights into the biological functions of IRGs.^9^ Metascape is a web portal for gene annotation and analysis. It provides automated meta‐analysis tool to understand common and unique pathways within a group of orthogonal target‐discovery studies. It also supports protein‐protein interaction (PPI) analysis based on BioGRID, interactive visualization of Gene Ontology (GO) Networks and enrichment heatmap generation.

### 2.4 Establishment of IRG signature with LASSO Cox regression model

The LASSO Cox regression analysis (LASSO, least absolute shrinkage and selection operator^10^) can achieve shrinkage and variable selection simultaneously by performing the Cox regression model with LASSO penalty. It was performed to establish the most valuable predictable IRG signature. Firstly, we used R software to generate a random number, and all samples in TCGA data sets were randomly assigned to the training cohort (n = 303) and the testing cohort (n = 101) in a ratio of 3:1 using the randomization method. Data set GSE13507 was set as the validation cohort. Univariable Cox analysis was used to identify immune‐related genes associated with survival. Then, LASSO Cox regression analysis was performed using R package ‘glmnet’ to construct a prognostic IRG gene signature based on survival‐related IRGs in the training group. We estimated the optimal values of penalty parameter lambda through 10‐fold cross‐validations.^11^, ^12^ A prognostic IRG signature was then constructed based on the mRNA expression level weighted by the estimated regression coefficient in the LASSO Cox regression model. The risk score of six‐IRG signature $=\sum_{i=1}^{n}\left(\text{coef}i*\text{Expr}i\right)$, where Expr*_i_* was the expression of the IRGs in the signature for patient *i*, and coef*_i_* is the LASSO coefficient of the IRGs i.

### 2.5 Estimate of IRG signature for patients' prognosis

The optimal cut‐off values of prognostic IRG signature were evaluated using the X‐tile software version 3.6.1 (Yale University School of Medicine, New Haven, CT, USA).^13^ The patients were separated into the high‐risk and low‐risk groups according to the optimal cut‐off values. Kaplan‐Meier survival analysis and log‐rank test were selected to assess the association between each IRG and survival of patients. The accuracy of the survival prediction based on the IRG signature was analysed by a time‐dependent receiver operating characteristic (ROC) curve,^14^ with quantification of the area under the curve at different cut‐off times using the ‘survival ROC’ package. Cox regression analyses and subgroup analyses were performed to identify whether six‐IRG signature was a clinically independent prognostic factor.

### 2.6 TIMER database analysis

TIMER is an online web database. It provides an open platform to explore, visualize and analyse the abundance of tumour‐infiltrating immune cells (TIICs) from gene expression profiles by a deconvolution previously published statistical method. According to TIMER database, we can analyse the correlation of IRG signature expression with the abundance of immune infiltrates, including B cells, CD8 T cells, CD4 T cells, macrophages, neutrophils and dendritic cells.

### 2.7 Construction and assessment of the nomogram

The nomogram was used to construct a prognostic scoring system for predicting survival in bladder cancer patient. In the nomogram, a vertical line was drawn from each risk factor to the ‘Points’ line to get a score in the nomogram. Then, the score of each risk factor was added to obtain the total points, which could be used to estimate survival probability. Calibration plots were performed to assess the performance of the nomogram. R package ‘rms’ was performed for construction and assessment of the nomogram.

### 2.8 Genetical alteration of IRG genes

The cBioPortal for Cancer Genomics (http://www.cbioportal.org/) is a large‐scale cancer genomics database.^15^ It provides an open platform to explore, visualize and analyse multi‐dimensional cancer genomic data. Researchers can interactively explore the genetic changes of different samples, genes and paths. This site also provides gene‐level graphical summaries from multi‐platform, web visualization analysis and survival analysis. We used cBioPortal to explore genetic alterations connected with the six IRGs and their correlation with other famous genes.

### 2.9 Gene set enrichment analysis (GSEA)

GSEA (http://software.broadinstitute.org/gsea/index.jsp) was used to explore biological function of six‐IRG signature.^16^ Annotated gene sets c2.cp.kegg. v5.2.symbols.gmt were chosen as the reference gene sets. Gene size ≥ 10, FDR < 0.05 and |enrichment score (ES)|> 0.65 were set as the cut‐off criteria.

### 2.10 Statistical analysis

R software version 3.5.2 was used for all the statistical analyses. All statistical tests were two‐sided, and *P* < .05 was considered statistically significant. Group comparisons were performed using the *t* test for continuous variables and chi‐squared test for categorical variables. The Harrell's concordance index (C‐index) and Akaike information criterion (AIC) were performed to measure and compare predictive accuracy of the prognostic models.

## 3 RESULTS

### 3.1 Identification of immune‐related genes and pathway analysis

**FIGURE 1:**
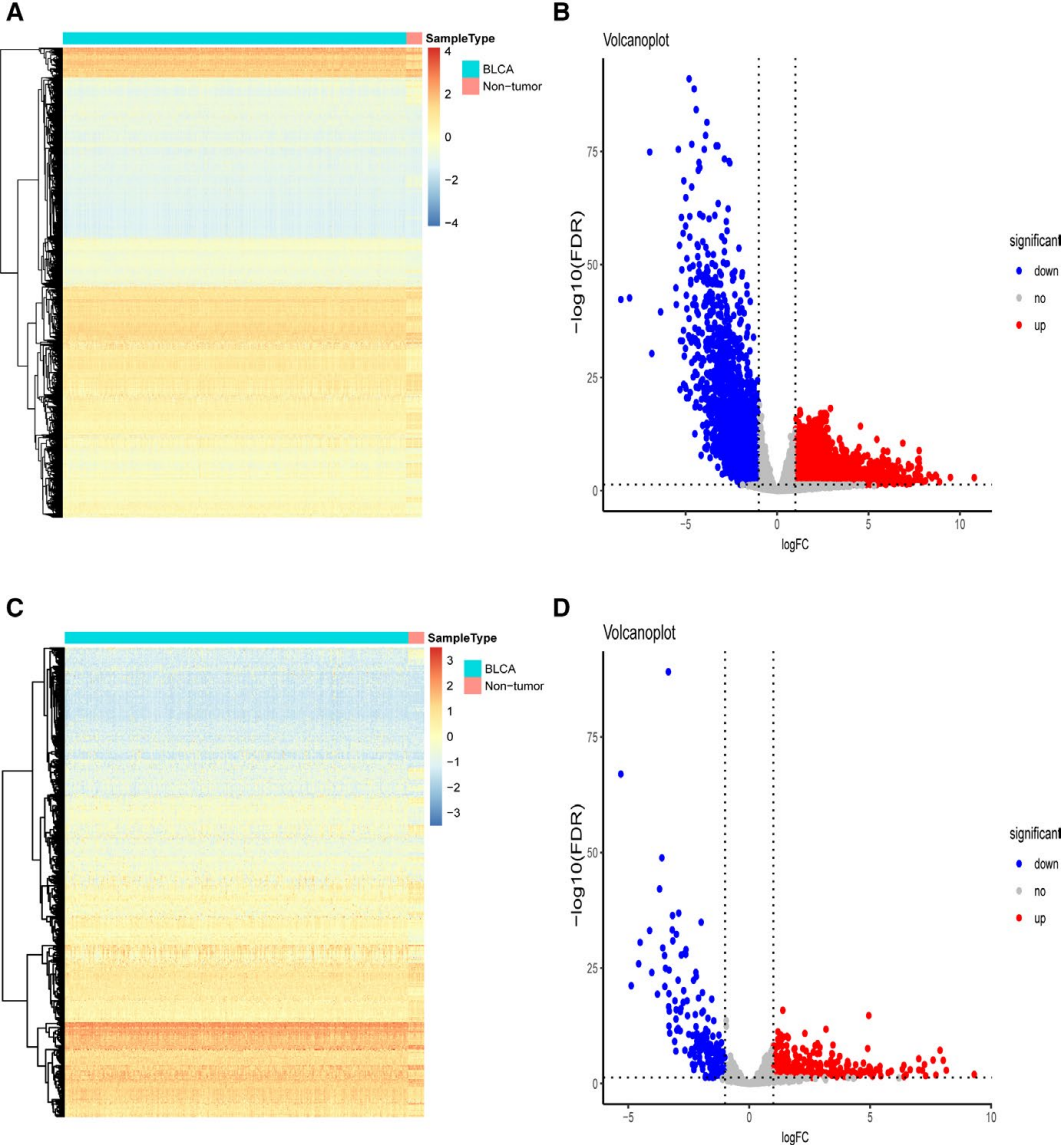
Differentially expressed immune‐related genes. A‐C, The heatmaps visualize the differentially expressed genes in TCGA data set and differentially expressed immune‐related genes (IRGs), respectively. B‐D, The volcano plots visualize the differentially expressed genes in TCGA data set and differentially expressed immune‐related genes (IRGs), respectively. The red nodes represent up‐regulated genes. The green nodes represent down‐regulated genes

**FIGURE 2:**
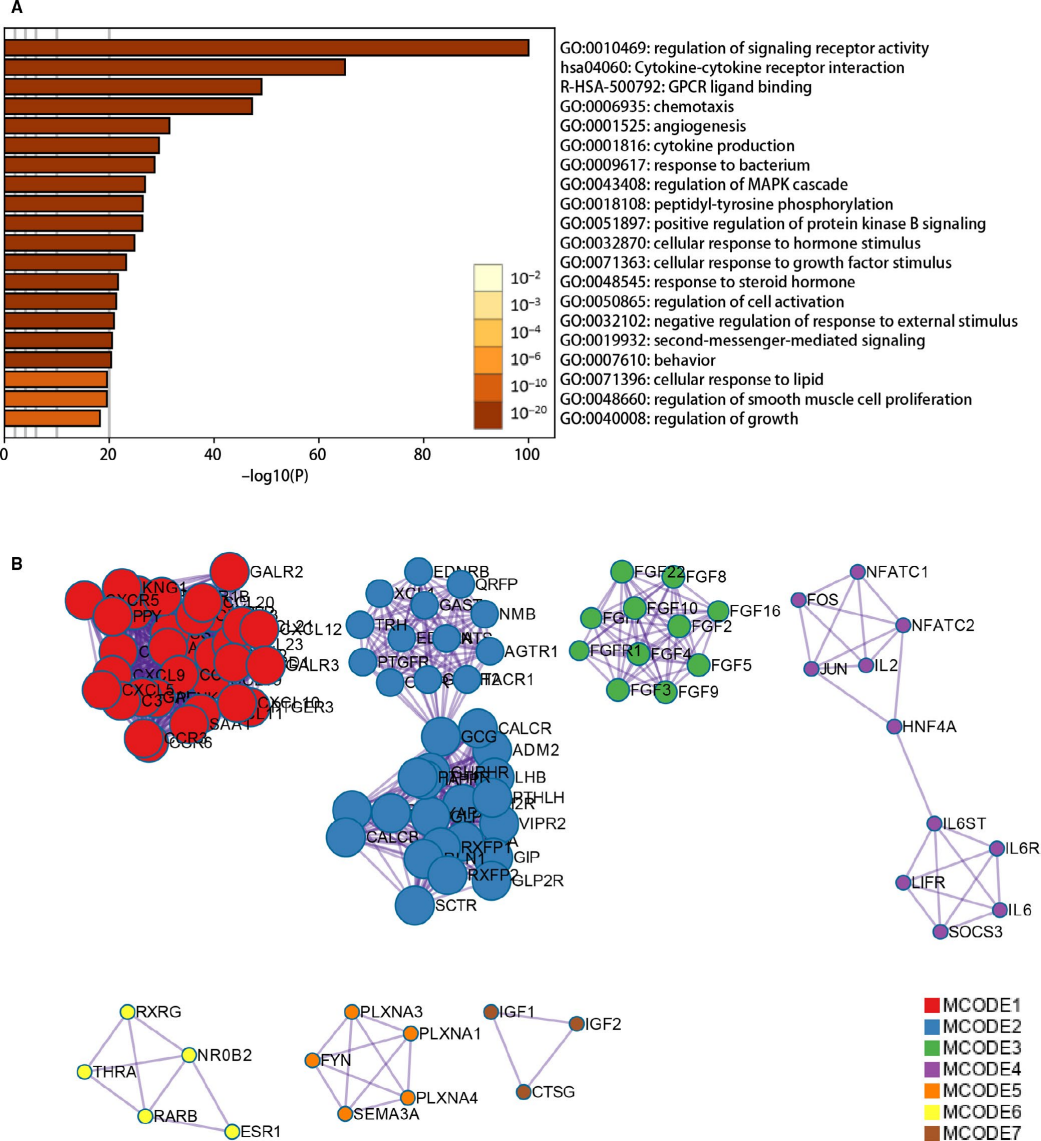
Functional enrichment analysis of differentially expressed immune‐related genes. A, Heatmap of the top 20 pathway and functional enrichment clusters, each bar represents a cluster. B, The 7 MCODE of all IRGs

The gene expression matrix, which consists of 414 bladder cancer specimens and 19 adjacent non‐tumour bladder specimens in the TCGA data set, was analysed to identify differentially expressed genes (DEGs). A total of 5180 DEGs were screened out with a threshold criterion |log2FC| > 1 and FDR < 0.05, including 3108 up‐regulated and 2072 down‐regulated. From this set of genes, we extracted 392 differentially expressed immune‐related genes (IRGs), including 215 up‐regulated and 177 down‐regulated genes. Heatmaps were performed to represent the unsupervised clustering of all the DEGs and IRGs (Figure 1A,C). Volcano plots were used to show the significantly DEGs and IRGs between bladder cancer tumour samples and adjacent non‐tumour samples (Figure 1B,D). Protein‐protein interaction network analysis from Metascape showed these genes were gathered in seven MCODE components (Figure 2B and Table S1). As expected, pathway and functional enrichment analyses showed that inflammatory and immune‐related pathways were most frequently implicated, including in ‘regulation of signaling receptor activity’, ‘cytokine‐cytokine receptor interaction’ and ‘GPCR ligand binding’ (Figure 2A).

### 3.2 Identification of the predictive six‐IRG signature for survival prediction

**FIGURE 3:**
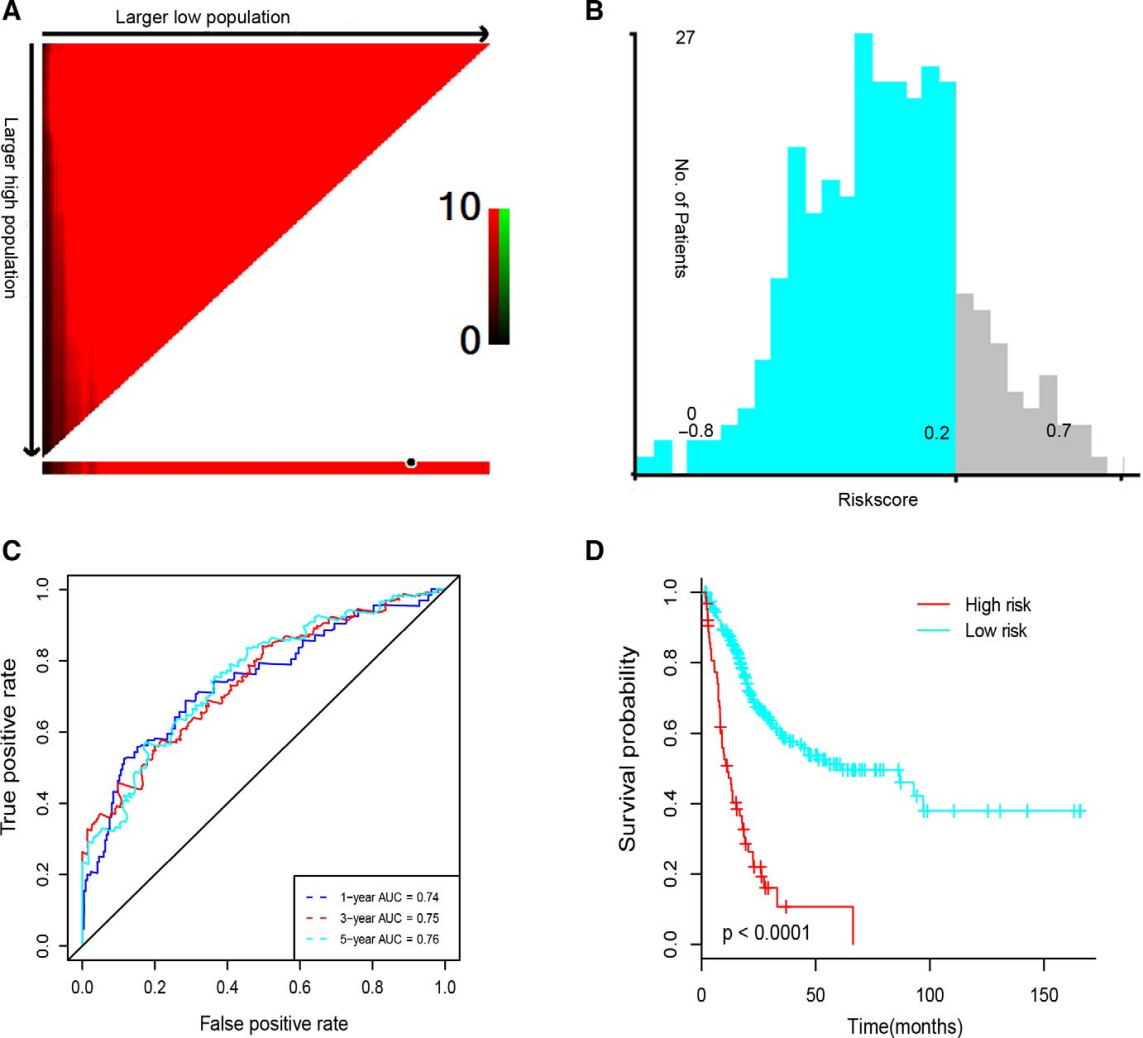
Prognostic analysis of six‐IRG signature in the training cohort. (A) X‐tile analysis indicates red inverse association between risk score and overall survival, (B) shows distribution of risk score of six‐IRG signature, (C) time‐dependent ROC for survival prediction models, (D) indicates Kaplan‐Meier survival curves between the high‐risk group and the low‐risk group

To avoid the interference of unrelated causes of death, patients with follow‐up time <30 days were excluded. Afterwards, by performing univariable Cox regression analysis between 392 differentially expressed IRGs above and survival data of 404 patients, we obtained 92 prognosis‐related IRGs, which all reached a statistical significance (*P* < .05). Then, all the 404 patients were randomly assigned to the training cohort (n = 303) and the testing cohort (n = 101). To precisely construct a prognostic model to predict the survival of bladder cancer patients, LASSO Cox regression model was performed to identify the most optimal prognostic IRG signature of bladder cancer patients in the training cohort (Figure S1). As the result, a prognostic IRG signature consisted of six immune‐related genes was screened out, including AHNAK, OAS1, APOBEC3H, SCG2, CTSE and KIR2DS4. Then, TIMER,^17^, ^18^ a software calculating the proportion of cell types, was performed to estimate the correlation between six IRGs and immune cell infiltration. As expected, the proportion of all these seven cell types was significantly and negatively correlated with six IRGs (Figure S2). Taken together, these results indicated that the six IRGs reflected the microenvironment of bladder cancer, especially immune cell infiltration. Next, the risk score was calculated as the following, the risk score of six‐IRG signature = (AHNAK*0.1036‐OAS1*0.114−APOBEC3H*0.0128+SCG2*0.0073−CTSE*0.0081‐KIR2DS4*0.0614). The optimal cut‐off value of the risk score was identified as 0.2 by X‐tile software (Figure 3A,B); then, all the patients were separated into the high‐risk group and the low‐risk group. The Kaplan‐Meier survival analysis demonstrated that patients in the high‐risk group (risk score > 0.2) were correlated with a trend towards worse survival compared with patients in the low‐risk group (risk score < 0.2) (Figure 3D). The prognostic accuracy of six‐IRG signature was assessed at the time‐points 1, 3 and 5 years using time‐dependent ROC analysis. The area under the curves (AUCs) were 0.74, 0.75 and 0.76, respectively (Figure 3C).

### 3.3 Validation of the six‐IRG signature for survival prediction

**FIGURE 4:**
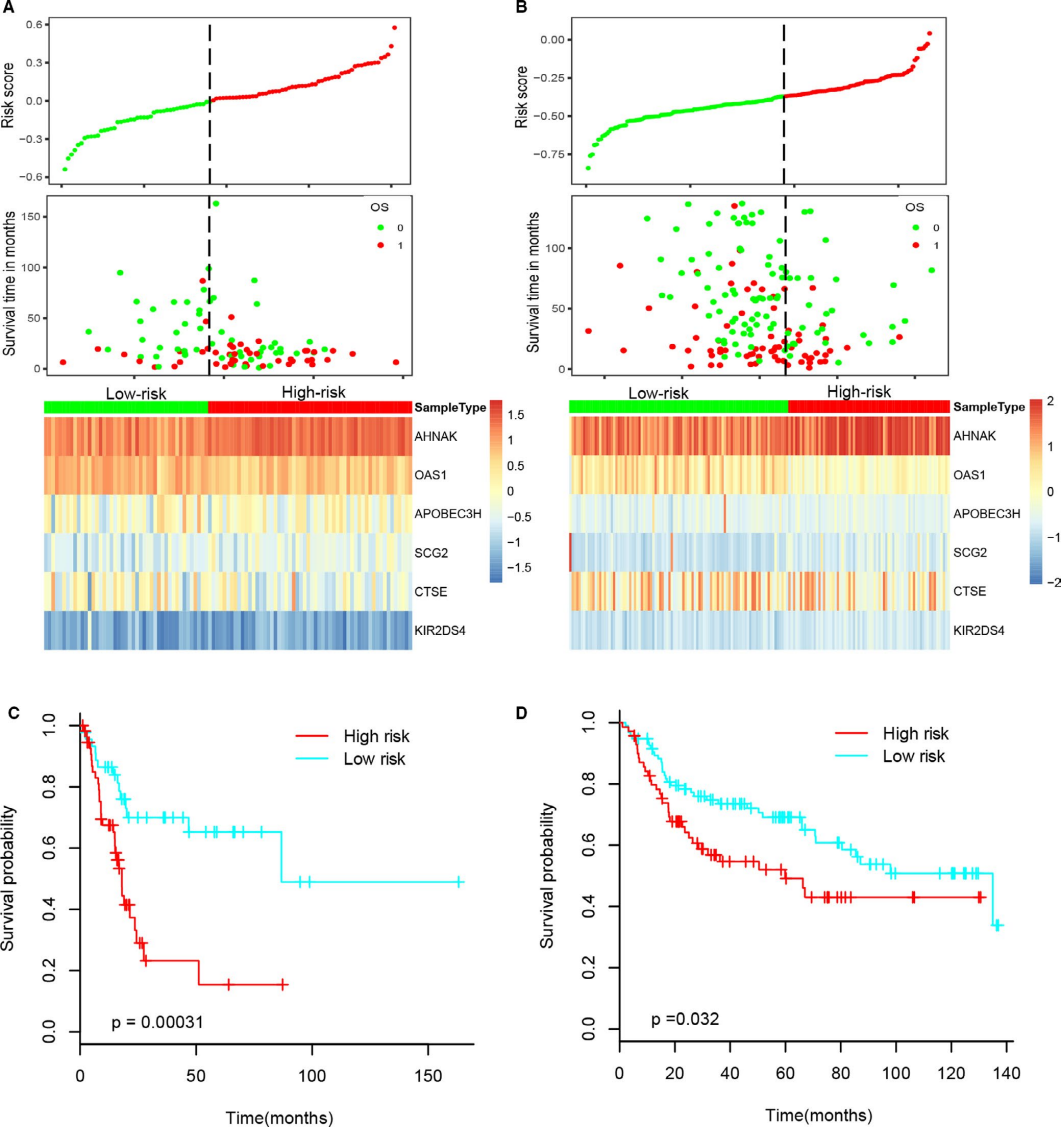
Prognostic analysis of six‐IRG signature in the testing cohort and the validation cohort. (A‐B) The distribution of risk score, OS and OS status and the heatmap of prognostic six‐IRG signature in the testing cohort and the validation cohort, respectively. The dotted line indicates the cut‐off point of the optimal risk score used to stratify patients into the low‐risk group and high‐risk group, (C‐D) the survival analysis of six‐IRG signature in the testing cohort and the validation cohort. Patients in the high‐risk group suffered shorter survival intervals

The prognostic value of six‐IRG signature was further assessed in the testing cohort and the validation cohort. Patients were assigned to the low‐risk group and the high‐risk group via the optimal cut‐off value of risk score in these two independent cohorts. The distributions of the six‐IRG signature based on risk score, survival status and six‐IRG expression profiles of testing set and validation set are shown in Figure 4A,B. Patients in the high‐risk groups showed poorer prognoses, whereas patients in the low‐risk groups had more favourable prognoses. Then, the results of Kaplan‐Meier survival curves were also consistent with Figure 4A,B.

### 3.4 Association of six‐IRG signature with patients' clinicopathological characteristics

To explore association between six‐IRG signature and patients' clinicopathological characteristics, clinicopathological data including age, gender, stage and grade were collected from TCGA database. All the patients were assigned to the high‐risk group and the low‐risk group based on the risk score cut‐off value of six‐IRG signature. We then analysed the relationship of six‐IRG signature and clinicopathological characteristics in bladder cancer patients. As shown in Table 1, the risk score of six‐IRG signature showed a significant relevance with age, stage and grade (*P* < .05). However, there was no association between the risk score and gender of patients with bladder cancer.

**Table 1:** Association of six‐IRG signature with clinicopathological characteristics

Variable	Total patients	Risk score		*P*
	No (%)	Low group	High group	
Age, mean ± SD (y)	67.0 ± 10.95	67.0 ± 10.95	71.16 ± 9.29	.003
Age				
<65	116 (38.3)	101 (87.1)	15 (12.9)	.006
≥65	187 (61.7)	138 (73.8)	49 (26.2)	
Gender				
Male	217 (71.6)	176 (81.1)	41 (18.9)	.16
Female	86 (28.4)	63 (73.3)	23 (26.7)	
Stage				
Ⅰ	3 (1.0)	3 (100)	0 (0)	<.001
Ⅱ	94 (31.1)	87 (92.6)	7 (7.4)	
Ⅲ	106 (35.1)	82 (77.4)	24 (22.6)	
Ⅳ	99 (32.8)	66 (66.7)	33 (33.3)	
Grade				
Low	14 (4.7)	14 (100)	0 (0)	.046
High	287 (95.3)	223 (77.7)	64 (22.3)	

### 3.5 Validation of the six‐IRG signature as an independent prognostic factor

**FIGURE 5:**
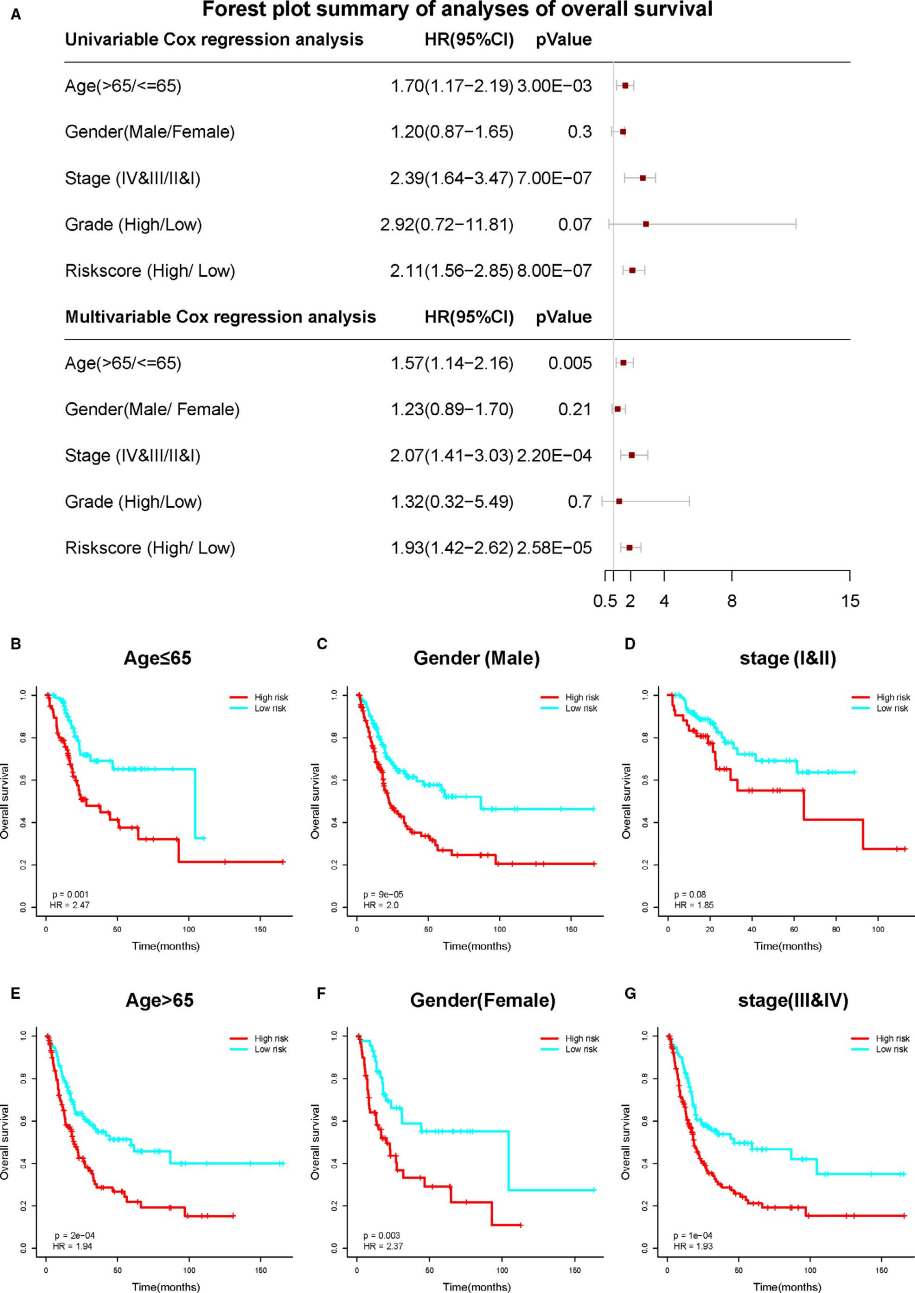
Forest plot summary and subgroup analysis of six‐IRG prognostic signature. A, Unavailable and multivariable analyses of overall survival in bladder cancer patients. The red squares on the transverse lines represent the hazard ratio (HR), and the grey transverse lines represent 95% CI. B‐G, Subgroup analysis of six‐IRG signature predicting survival by stratifying patients with various clinicopathological risk factors. The patients are stratified into six subgroups based on (A) age > 65, (B) age ≤ 65, (C) gender (male), (D) gender (female), (E) stages I & II and (F) stages III & IV

To explore whether six‐IRG signature was a clinically independent prognostic factor in bladder cancer patients, we performed univariable and multivariable Cox regression analyses in TCGA data set. The risk score of six‐IRG signature and other clinicopathological variables, including age, gender, stage and grade, were included as covariates. The result is demonstrated in Figure 5A, the six‐IRG signature remained to be an independent prognostic factor even adjusted by age and other covariates in multivariable analyses.

### 3.6 Subgroup analysis of six‐IRG signature for survival prediction

To evaluate the prognostic value of six‐IRG signature within the same clinicopathological risk factors, we further performed subgroup analysis. The patients were stratified into different subgroups, including younger group (age ≤ 65), elder group (age > 65), male group, female group, earlier‐stage group (stages I & II) and advanced‐stage group (stages III & IV). The results revealed that the six‐IRG signature was quite useful in nearly all subgroups, with clinically and statistically significant prognostic value (Figure 5B‐G).

### 3.7 Construction of nomogram prognostic model based on six‐IRG signature

**FIGURE 6:**
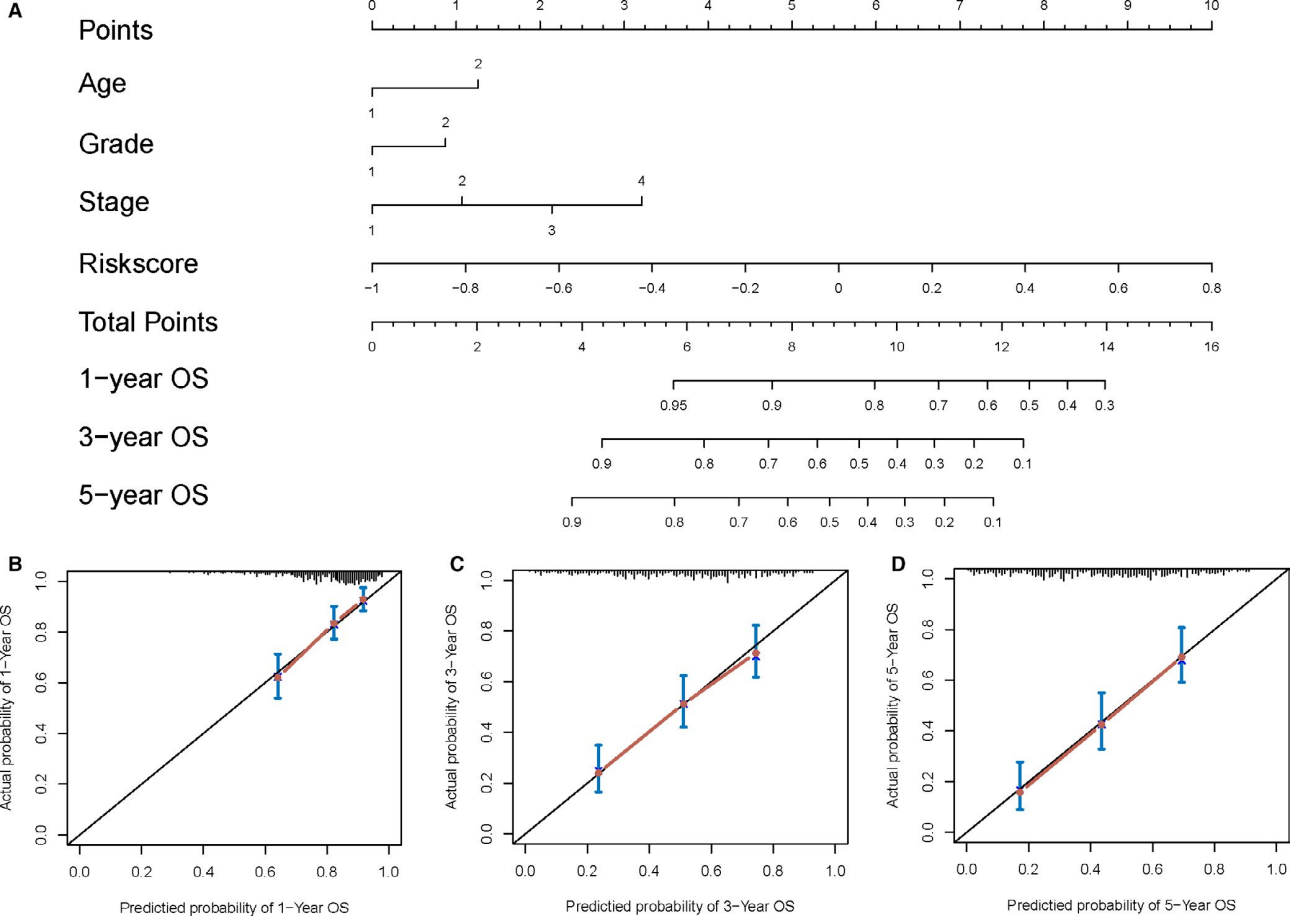
Nomogram, calibration plots and clinical impact plots for the prediction of OS survival in bladder cancer patients. A, Nomogram for the prediction of OS at 1, 3 and 5 y. B‐D, The calibration plots for predicting OS at 1, 3 and 5 y, diagonal line: ideal model; vertical bars: 95% confidence interval

As six‐IRG signature performed a strong prognostic ability, we explored its potential to improve prognostic accuracy of bladder cancer clinicopathological risk factors. The result showed the accuracy and efficiency of prognostic risk factors for bladder cancer including age, grade and stage were improved, after incorporating six‐IRG signature (Table S2). The integration of six‐IRG signature and other prognostic risk factors showed higher c‐indices and lower AICs than a single prognostic risk factor. Hence, we established a novel nomogram model for predicting clinical outcome in patients with bladder cancer. According to nomogram total points of all risk factors, we could provide an individualized risk prediction for bladder cancer patients accurately (Figure 6A). For example, a 72‐year‐old patient diagnosed with high‐grade bladder cancer (T2N0M0), with IRG score equal to 0, the total points of this patient are 1.25 + 0.8 + 1.1 + 5.6 = 8.75. According to the nomogram, the predicted 1‐year OS is approximately 85%, 3‐year OS is approximately 57%, whereas 5‐year OS is approximately 49%. The calibration plots showed nomogram did well compared with the ideal model for predicting OS at 1, 3 and 5 years (Figure 6B‐D)

### 3.8 Gene set enrichment analysis (GSEA)

**FIGURE 7:**
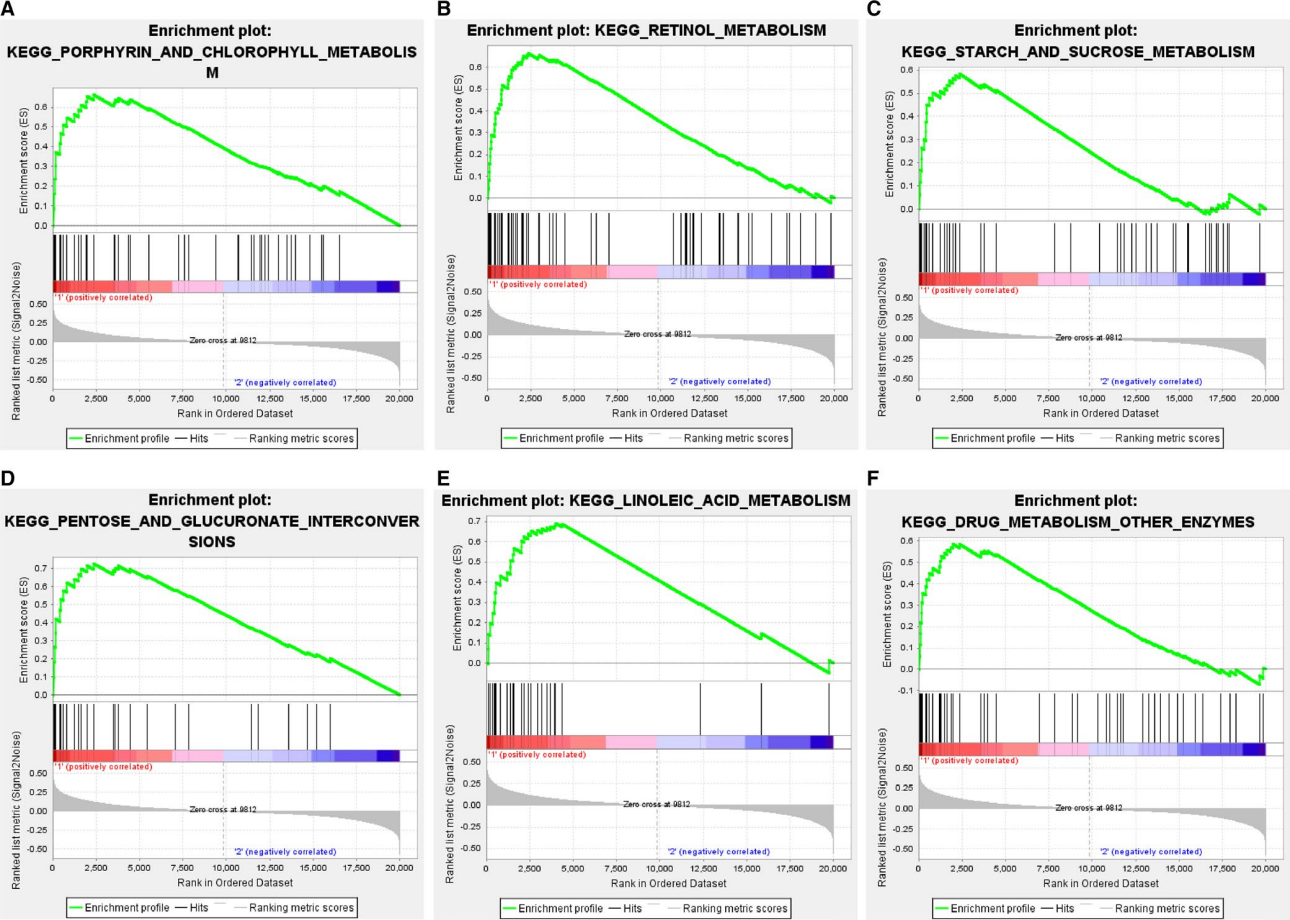
Gene set enrichment analysis (GSEA). Only listed the common functional gene sets enriched in six‐IRG signature highly expressed group

Gene set enrichment analysis software was performed to investigate the potential biological significance of key genes in six‐IRG signature. The results revealed that high expression samples in all six key genes were enriched in porphyrin and chlorophyll metabolism, retinol metabolism, starch and sucrose metabolism, pentose and glucuronate interconversions, linoleic acid metabolism, drug metabolism other enzymes (Figure 7A‐F).

### 3.9 Genetical alteration of hub genes

The alteration statuses of six IRGs were analysed using TCGA bladder cancer patients' data of cBioPortal database. The six IRGs altered in 122 (29.5%) of 413 cases (Figure S3B), and the frequency of alteration of each hub gene is shown in Figure S3A. APOBEC3H and OAS1 altered most (10% and 6%, respectively). mRNA up‐regulation and amplification were the main type. Figure S3C demonstrates the relationship of the 9 genes and the other 50 most frequently altered neighbour genes. OAS1 was significantly important in the network.

### 3.10 Distribution of IRG scores in different molecular subtypes

**FIGURE 8:**
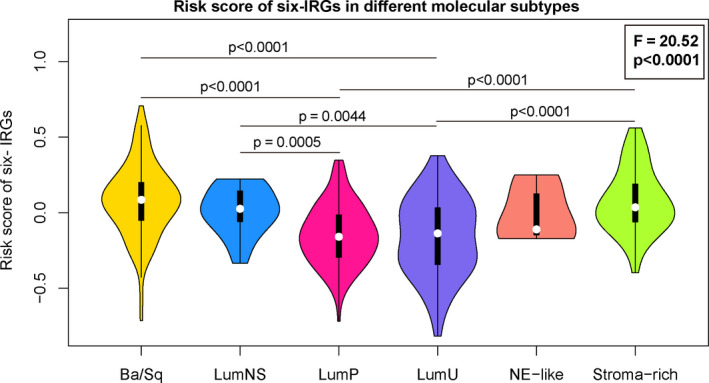
Risk score of six IRGs in six consensus molecular subtypes

Bladder cancer is a heterogeneous disease that is characterized by genomic instability and high gene mutation rate. According to different genomic and transcriptomic characteristics, bladder cancer can be classified into different molecular subtypes.^19^ Different molecular subtypes had dramatically different clinical prognosis and respond variably to therapeutic options. To explore the relationship between IRG scores and molecular subtypes, we analysed the distribution of IRG scores in different consensus molecular subtypes, and the results showed the IRG scores had significant difference in different molecular subtypes (Figure 8). The Ba/Sq subtype with the poor prognosis had the highest IRG scores, but the best prognostic LumU and LumP also had low IRG scores.

## 4 DISCUSSION

Recently, the clinical application of immunosuppressive point inhibitors is one of the most important advances in the field of cancer treatment. But immune evasion mechanisms in the TME of advanced human cancers are highly heterogeneous. The PD‐1/PDL1 pathway is responsible for dysfunctional immunity in fewer than 40% of human solid tumours.^20^, ^21^ Except for the PD‐1/PD‐L1, many other molecular or cellular mechanisms also contribute to dysfunctional immunity in the TME, for example up‐regulation of suppressive molecules, cytokines, metabolites and down‐regulation of immune stimulatory molecules.^22^, ^23^ Korpal et al found PPARγ/RXRα pathway impairs CD8^+^ T cell infiltration and confers partial resistance to immunotherapies of bladder cancer.^24^ A study based on 209 patients with bladder cancer showed immune‐inflammation index predicts prognosis of bladder cancer patients after radical cystectomy.^25^ Luo et al applied the Estimation of STromal and Immune cells in MAlignant Tumors using Expression data (ESTIMATE) algorithm to identify 27 tumour microenvironment‐related genes of bladder cancer, these genes are associated with poor prognosis of patients with bladder cancer and are the potential new therapeutic target for bladder cancer.^26^ But currently bladder cancer tumour immune mechanism is not yet clear. Thus, this study investigated the immunogenomic landscape of bladder cancer based on large‐scale open bioinformatics data resources and then developed an IRG‐based prognostic gene signature for bladder cancer patients.

In this study, we identified differentially expressed immune‐related genes in bladder cancer patients according to genome‐wide profiling analysis. On the basis of the pathway and functional enrichment analyses, the differentially expressed immune‐related genes may play crucial roles in regulation of signalling receptor activity, cytokine‐cytokine receptor interaction, GPCR ligand binding, chemotaxis, angiogenesis, cytokine production and MAPK signalling pathway. Then, the immune‐related genes correlated with survival of bladder cancer patients were selected by Kaplan‐Meier survival analysis. Next, based on LASSO Cox regression, an immune‐related prognostic gene signature was constructed, which was significantly associated with OS in bladder cancer patients.

The prognostic gene signature consists of six immune‐related genes, AHNAK, OAS1, APOBEC3H, SCG2, CTSE and KIR2DS4. AHNAK is a protein with a molecular mass of 700 KDa, and it is widely distributed in many types of cells. Some researchers have also discovered that the AHNAK gene is differently expressed in various human malignancies and abnormal expression of AHNAK may be related to the invasion and metastasis of tumour cells. Dumitru CA *et al* found combination high levels of neutrophilic infiltration, macrophage migration inhibitory factor and AHNAK overexpression significantly associated with poor survival in laryngeal carcinoma,^27^ and they hypothesize that neutrophils might enhance tumour cell migration/invasion via AHNAK. OAS1 is an antiviral protein (AVP) induced by interferon, and it plays an important role in antiviral immunity by regulating multiple signalling pathways.^28^, ^29^ Latest research showed that the OAS1 could regulate the antitumour activity and toxicity of AZA and related drugs by OAS‐RNase L innate immune pathway.^30^ APOBEC3H is a member of the apolipoprotein B mRNA‐editing enzyme catalytic polypeptide 3 families of proteins, and it induces somatic mutagenesis in cancer cells that drive tumour evolution and may manifest clinically as recurrence, metastasis and/or therapy resistance.^31^ Hence, it may be used as target for cancer therapy. SCG2 is a neuroendocrine secretory proteins, and it can regulate leucocyte, endothelial and mesenchymal cell functions.^32^ Yon et al found malignant pheochromocytomas can secrete SCG2, and it can be used as a potential marker for progression of pheochromocytoma.^33^ CTSE can encode peptidases the A1 family. It plays an immune role by regulating antigen presentation and chemotaxis. The latest researches showed CTSE was high expressed in various cancers, and stimulated oncogenesis and progression.^34^, ^35^, ^36^ KIR2DS4 was a killer cell immunoglobulin‐like receptor, and it co‐ordinates with HIA class I allotypes to active natural killer cells and plays an important role in natural immunity. It also correlated with progression of various tumours.^37^, ^38^ Furthermore, we performed multivariable Cox regression and subgroup analysis to found this six‐IRG signature enabled to stratify patients into the low‐risk and high‐risk groups with distinct differences in survival of patients with bladder cancer. Then, a nomogram based on six‐IRG signature and other clinicopathological risk factors was constructed for clinical practice, and it performed well in predict patients' survival. Hence, this nomogram might be used as a prognostic tool for patients with bladder cancer in real‐life clinical practice and provided clinical reference for physicians in the decision‐making process. Moreover, we explored biological function of the six‐IRG signature. The results revealed mRNA up‐regulation and amplification were the main alteration type of six genes. GSEA showed these six immune‐related genes may induce carcinogenesis and progression through multiple metabolic pathways. Bladder cancer is also a heterogeneous cancer; different molecular subtypes have different clinical characteristics, respond variably to therapeutic options and have dramatically different prognosis; and we found IRG scores were dramatically different in different molecular subtypes, Therefore, our six‐IRG signature might be a vital tool for survival prediction in bladder cancer patients, aiding in personalized therapeutic treatment strategies and post‐operative counselling.

However, this study has some limitations. Firstly, all the data of this study were obtained from publicly available database. Some important clinical information was not available to us, for example, we cannot know about status of the patient's infection, whether the patients have used anti‐inflammatory drugs; these factors may bias the results of the experiment. Second, this is a retrospective study, and a multi‐centre and prospective study is needed to validate our results. Finally, further research is needed to elucidate molecular mechanisms of immune‐related genes.

In conclusion, the current study gained insight into the immunogenomic landscape of bladder cancer and identified a novel six‐IRG‐based prognostic model to predict survival of bladder cancer patients. The six‐IRG‐based nomogram had higher prognostic value than the conventional TNM stage in patients with bladder cancer. Therefore, our research may provide a novel immune‐related gene signature to estimate prognosis for patient survival with bladder cancer, and it might facilitate bladder cancer patients counselling and individualized management.

## CONFLICT OF INTEREST

The authors declare no conflict of interest.

## AUTHOR CONTRIBUTION

**Yongwen Luo:** Conceptualization (lead); Data curation (lead); Formal analysis (lead); Software (lead); Writing‐original draft (equal); Writing‐review & editing (equal). **Liang Chen:** Data curation (equal); Software (equal). **Qiang Zhou:** Investigation (equal); Validation (equal). **Yaoyi Xiong:** Formal analysis (equal); Investigation (equal). **Gang Wang:** Funding acquisition (equal); Methodology (equal). **Xuefeng Liu:** Methodology (equal). **Yu Xiao:** Writing‐original draft (equal); Writing‐review & editing (equal). **Lingao Ju:** Funding acquisition (equal); Writing‐review & editing (equal). **Xinghuan Wang:** Project administration (lead); Supervision (lead); Writing‐review & editing (equal).

## Supporting information

Fig S1‐S3

Tab S1

Tab S2

## Data Availability

The data that support the findings of this study are openly available in The Cancer Genome Atlas (TCGA) data portal (https://tcga‐data.nci.nih.gov/tcga/), Gene Expression Omnibus (GEO) database (http://www.ncbi.nlm.nih.gov/geo/) and ImmPort data portal *(* https://www.immport.org *)*.
