# Supplementary material for: Identification of a prognostic gene signature based on an immunogenomic landscape analysis of bladder cancer
Source: J Cell Mol Med. 2020 Oct 13;24(22):13370–82. doi: 10.1111/jcmm.15960 (PMC7701570; doi:10.1111/jcmm.15960)
Supplement: Supplementary file 1 — Fig S1‐S3 [file JCMM-24-13370-s001.docx]

Supplementary Figures

**
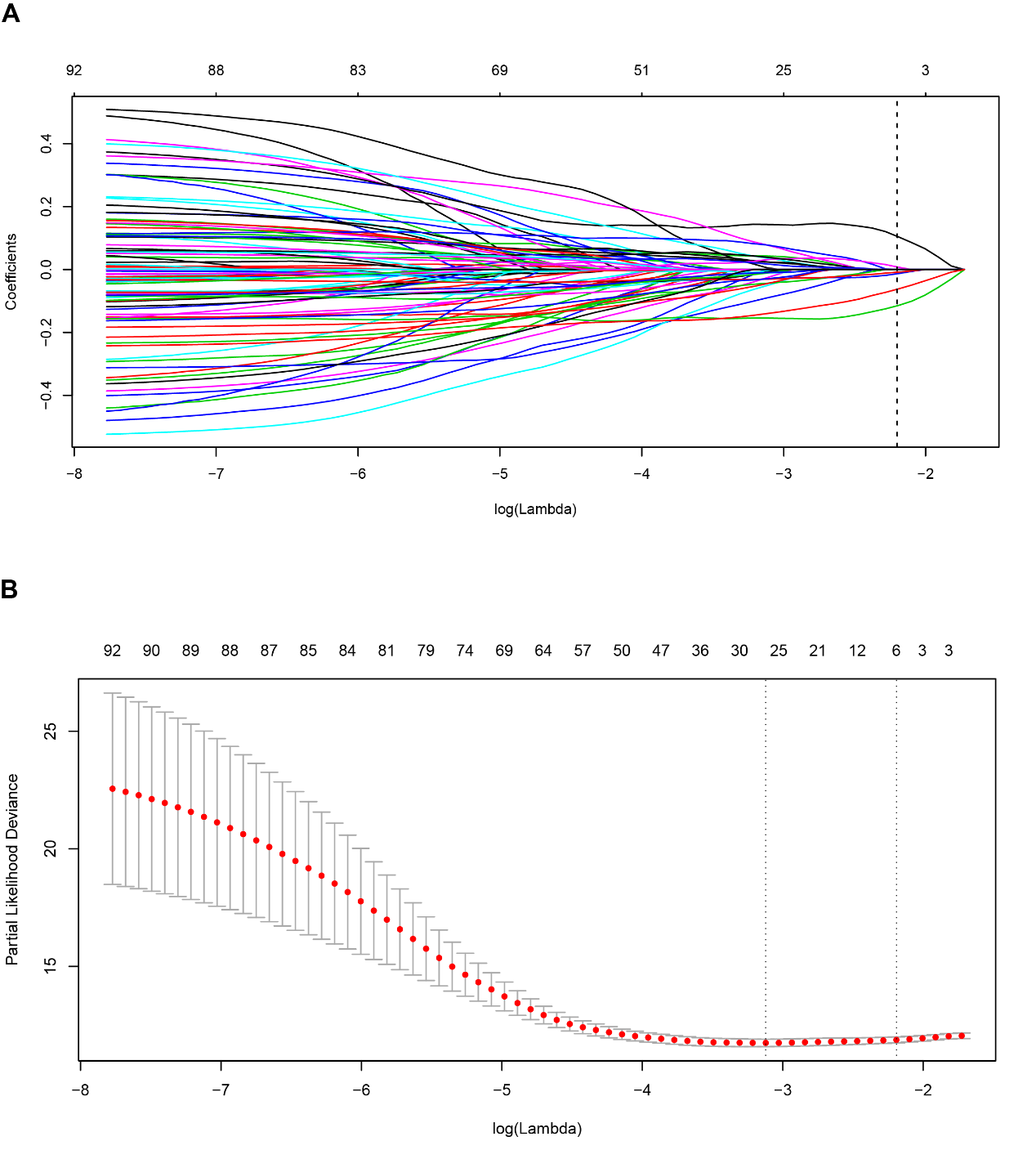
**

**Figure S1. Construction of the most valuable prognostic IRGs signature by LASSO Cox model.** **(A)** The LASSO coefficients profiles of six IRGs, a vertical line is drawn at the value chosen by 10-fold cross-validation. **(B)** Tuning parameter (Lambda, λ) selection cross-validation error curve. The vertical lines were drawn at the optimal values by the minimum criteria and the 1-SE criteria. We choose the right line by 1-SE criteria where the value = 0.0927468, with λ = 0.02767237.

**
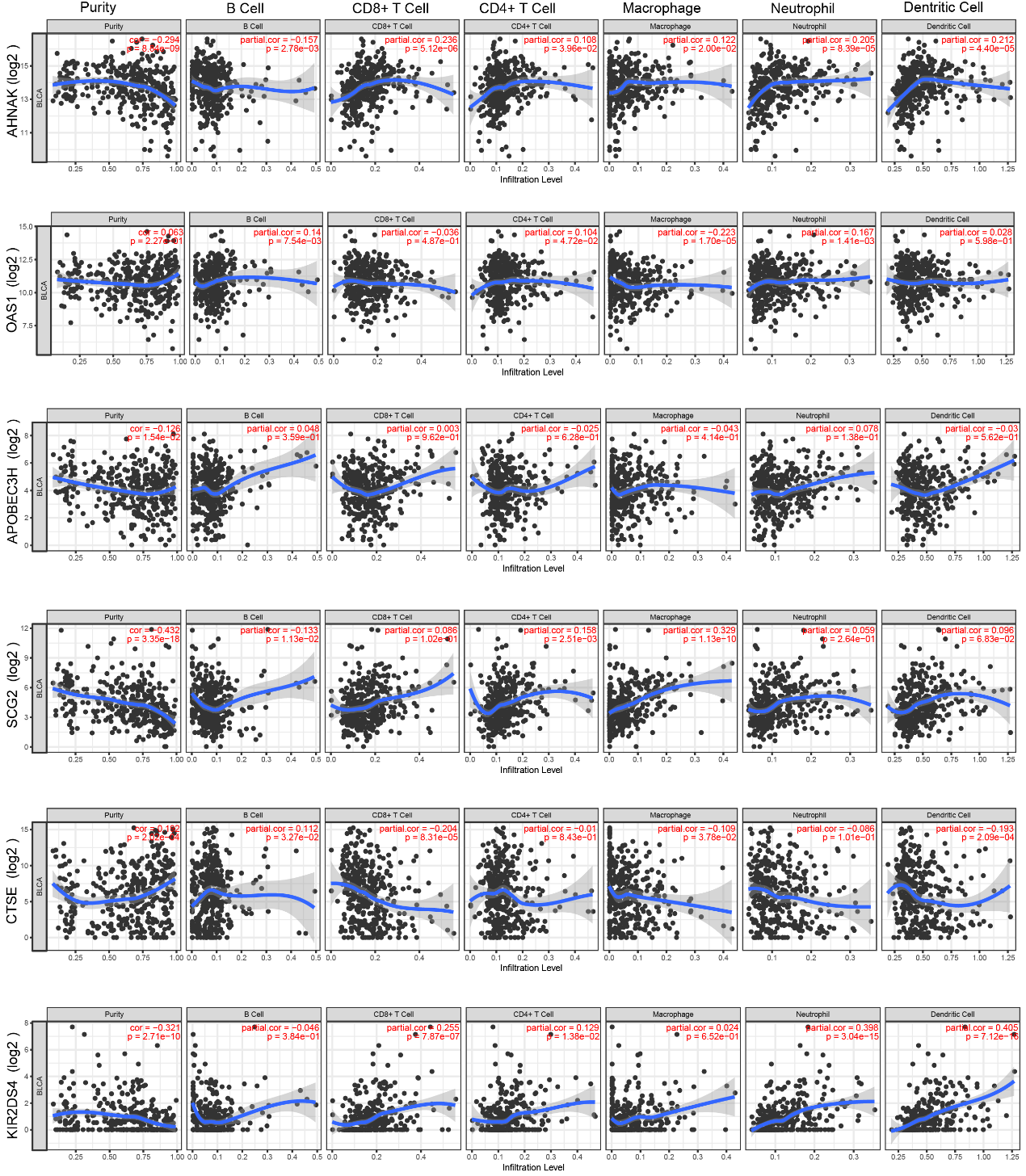
**

**Figure S2.** **Relationships between the six immune-related genes and infiltration abundances of six types of immune cells.** The correlation was performed by using Pearson correlation analysis. (**A**) Purity; (**B**) B cells; (**C**) CD8 T cells; (**D**) CD4 T cells; (**E**) macrophages; (**F**) neutrophils; and (**G**) dendritic cells.

**
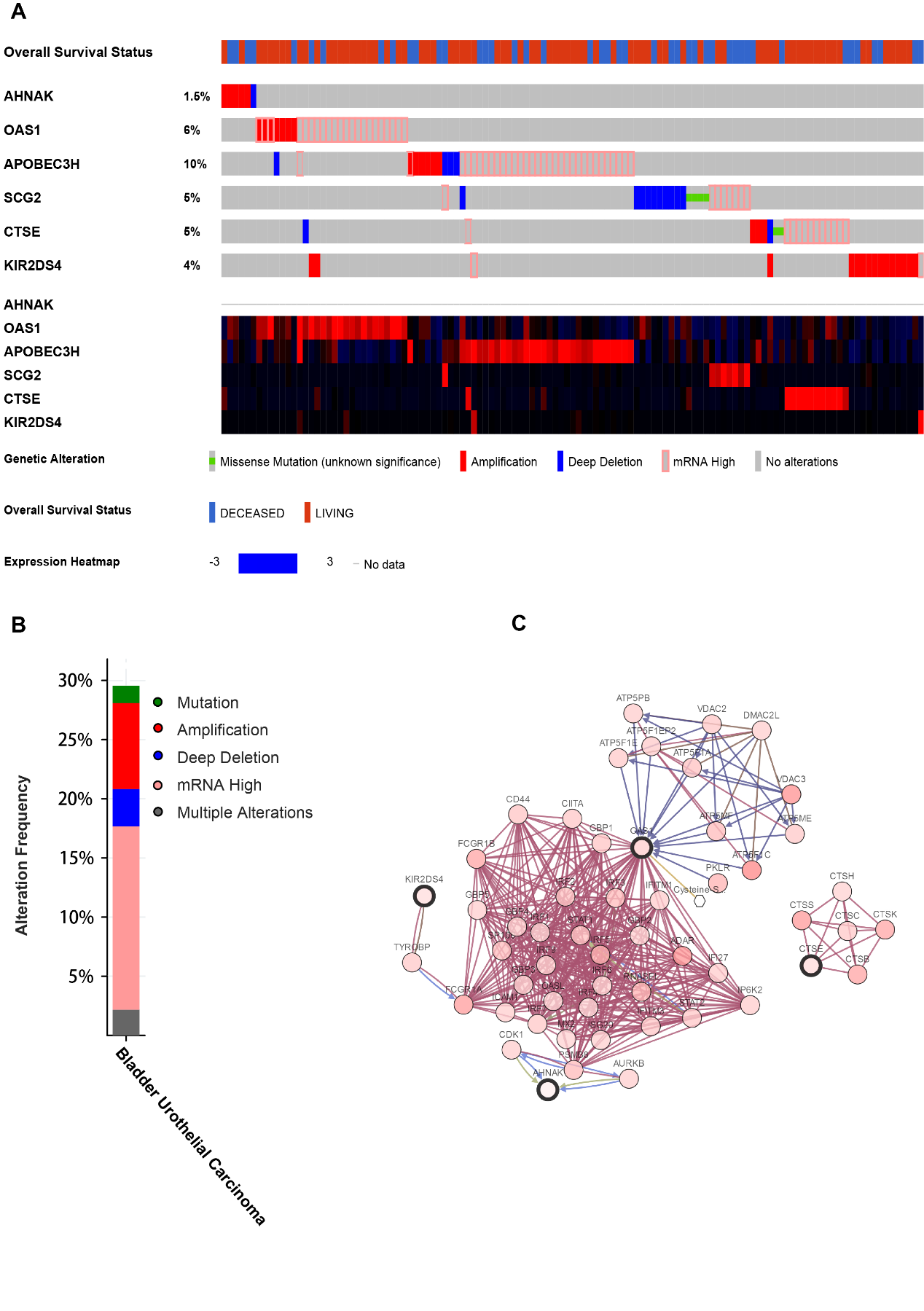
**

**Figure S3.** **Genetic alterations associated with 6 key genes. (A)** A visual summary of Genetic alterations (data from BLCA in TCGA, provisional) shows the genetic alteration of 6 key genes which were altered in 122 (29.5%) of 413 bladder cancer patients. **(B)** The total alteration frequency of 6 key genes is illustrated. **(C)** The network contains 56 nodes, including 6 key genes and the 50 most frequently altered neighbor genes. Relationship of 6 key genes is also illustrated.
