# Supplementary material for: Identification of a prognostic gene signature based on an immunogenomic landscape analysis of bladder cancer
Source: J Cell Mol Med. 2020 Oct 13;24(22):13370–82. doi: 10.1111/jcmm.15960 (PMC7701570; doi:10.1111/jcmm.15960)
Supplement: Supplementary file 2 — Tab S1 [file JCMM-24-13370-s002.docx]

| Network | Annotation |
| --- | --- |
| gene | GO:0010469\|regulation of signaling receptor activity\|-94.3;M5883\|NABA SECRETED FACTORS\|-68.0;M5885\|NABA MATRISOME ASSOCIATED\|-66.5 |
| gene_MCODE_ALL | R-HSA-500792\|GPCR ligand binding\|-89.6;R-HSA-373076\|Class A/1 (Rhodopsin-like receptors)\|-69.3;R-HSA-375276\|Peptide ligand-binding receptors\|-65.4 |
| gene_SUB1_MCODE_1 | R-HSA-373076\|Class A/1 (Rhodopsin-like receptors)\|-66.4;R-HSA-418594\|G alpha (i) signalling events\|-62.7;R-HSA-375276\|Peptide ligand-binding receptors\|-61.6 |
| gene_SUB1_MCODE_2 | R-HSA-500792\|GPCR ligand binding\|-52.9;hsa04080\|Neuroactive ligand-receptor interaction\|-30.0;R-HSA-373080\|Class B/2 (Secretin family receptors)\|-27.3 |
| gene_SUB1_MCODE_3 | R-HSA-2033519\|Activated point mutants of FGFR2\|-31.9;R-HSA-5654221\|Phospholipase C-mediated cascade FGFR2\|-31.6;R-HSA-190241\|FGFR2 ligand binding and activation\|-31.0 |
| gene_SUB1_MCODE_4 | hsa04659\|Th17 cell differentiation\|-16.7;M183\|PID IL6 7 PATHWAY\|-13.7;M167\|PID AP1 PATHWAY\|-12.7 |
| gene_SUB1_MCODE_5 | R-HSA-399955\|SEMA3A-Plexin repulsion signaling by inhibiting Integrin adhesion\|-16.5;R-HSA-399956\|CRMPs in Sema3A signaling\|-16.2;R-HSA-399954\|Sema3A PAK dependent Axon repulsion\|-16.2 |
| gene_SUB1_MCODE_6 | R-HSA-383280\|Nuclear Receptor transcription pathway\|-13.5;GO:0006367\|transcription initiation from RNA polymerase II promoter\|-10.6;GO:0043401\|steroid hormone mediated signaling pathway\|-10.5 |
| gene_SUB1_MCODE_7 | R-HSA-381426\|Regulation of Insulin-like Growth Factor (IGF) transport and uptake by Insulin-like Growth Factor Bi\|-6.9;GO:0040008\|regulation of growth\|-4.6;M5885\|NABA MATRISOME ASSOCIATED\|-4.5 |
