# Supplementary material for: Identification of a prognostic gene signature based on an immunogenomic landscape analysis of bladder cancer
Source: J Cell Mol Med. 2020 Oct 13;24(22):13370–82. doi: 10.1111/jcmm.15960 (PMC7701570; doi:10.1111/jcmm.15960)
Supplement: Supplementary file 3 — Tab S2 [file JCMM-24-13370-s003.docx]

**Table S2. Comparison of the predictive accuracy of the prognostic models.**

| **Model** | **Overall survival** | |
| --- | --- | --- |
|  | **C-index** | **AIC** |
| Risk score | 0.661±0.023 | 1862.96 |
| Age | 0.567±0.019 | 1917.24 |
| Grade | 0.515±0.005 | 1919.87 |
| Stage | 0.63±0.021 | 1877.31 |
| Risk score+Age+Grade+Stage | 0.71±0.02 | 1826.89 |
